# Establishment of an in vitro chicken epithelial cell line model to investigate *Eimeria tenella* gamete development

**DOI:** 10.1186/s13071-018-2622-1

**Published:** 2018-01-18

**Authors:** Françoise I. Bussière, Alisson Niepceron, Alix Sausset, Evelyne Esnault, Anne Silvestre, Robert A. Walker, Nicholas C. Smith, Pascale Quéré, Fabrice Laurent

**Affiliations:** 1 ISP, INRA, Université François Rabelais de Tours, UMR 1282, 37380 Nouzilly, France; 2 Institute of Parasitology, University of Zurich, Winterthurerstrasse 266a, CH-8057 Zurich, Switzerland; 3 Research School of Biology, Australian National University, Canberra, ACT 2601 Australia

**Keywords:** *Eimeria tenella*, Chicken epithelial cells, Macrogametes, Microgametes

## Abstract

**Background:**

*Eimeria tenella* infection leads to acute intestinal disorders responsible for important economic losses in poultry farming worldwide. The life-cycle of *E. tenella* is monoxenous with the chicken as the exclusive host; infection occurs in caecal epithelial cells. However, in vitro, the complete life-cycle of the parasite has only been propagated successfully in primary chicken kidney cells, which comprise undefined mixed cell populations; no cell line model has been able to consistently support the development of the sexual stages of the parasite. We therefore sought to develop a new model to study *E. tenella* gametogony in vitro using a recently characterised chicken cell line (CLEC-213) exhibiting an epithelial cell phenotype.

**Methods:**

CLEC-213 were infected with sporozoites from a precocious strain or with second generation merozoites (merozoites II) from wild type strains. Sexual stages of the parasite were determined both at the gene and protein levels.

**Results:**

To our knowledge, we show for the first time in CLEC-213, that sporozoites from a precocious strain of *E. tenella* were able to develop to gametes, as verified by measuring gene expression and by using antibodies to a microgamete-specific protein (EtFOA1: flagellar outer arm protein 1) and a macrogamete-specific protein (EtGAM-56), but oocysts were not observed. However, both gametes and oocysts were observed when cells were infected with merozoites II from wild type strains, demonstrating that completion of the final steps of the parasite cycle is possible in CLEC-213 cells.

**Conclusion:**

The epithelial cell line CLEC-213 constitutes a useful avian tool for studying *Eimeria* epithelial cell interactions and the effect of drugs on *E. tenella* invasion, merogony and gametogony.

## Background

Coccidiosis has a high economic impact on poultry farming worldwide [1]. This disease is caused by species of *Eimeria*, a genus of obligate intracellular parasites belonging to the phylum Apicomplexa; *Eimeria* spp. invade and multiply in intestinal epithelial cells [2]. *Eimeria tenella* is one of the most pathogenic species infecting chickens [3]. Its life-cycle is comprised of endogenous asexual multiplication and sexual development. Asexual multiplication consists of - typically, for wild type strains - three rounds of merogony, resulting in successive generations of schizonts, containing merozoites. This step is followed by sexual development (gametogony) with the formation of microgametes (mature male gametocytes) and macrogametes (mature female gametocytes) [4]. Each microgametocyte produces approximately 100 motile microgametes that are able to fertilise a macrogamete resulting in the formation of a zygote. The latter becomes encapsulated in a protective wall, forming an oocyst that is released from the host into the external environment. The precocious strain develops with a shortened prepatent period and presents only one round of merogony before entering gametogony. This strain is currently used for immunization as its pathogenicity is attenuated [4–6]. In vitro cell cultures permit limited parasite development, often with cessation of development at the first generation of merozoites (merozoites I) [7–11]. Macrogametes, microgametes and oocysts have only been observed in primary cultures of chick embryonic kidney cells or chicken kidney cells (PCKC), but oocyst yields remain consistently low [7, 12]. However, given that *Eimeria* spp. are highly host-specific [13], it may be possible to develop an in vitro assay based on chicken epithelial cell lines to study sexual stages of *E. tenella* life-cycle, host-pathogen interactions and large-scale drug screening.

In the first part of this study, we compared the ability of *E. tenella* to invade and develop to first generation schizonts in different cell lines. We then tested: (i) a commonly used cell line for *E. tenella* studies, Madin-Darby bovine kidney (MDBK) epithelial-like cells; (ii) a transimmortalized mouse intestinal epithelial cells (m-IC_cl2_), since the digestive tract is the site of *Eimeria* infection; and (iii) the chicken lung epithelial cell line (CLEC-213), cells of which are polarized, develop junctional complexes, express the cell to cell adhesion molecule, E-cadherin, and exhibit microvilli at the apical cell surface [14]. We then reasoned that, since in the literature, there are no chicken intestinal or caecal epithelial cell lines characterised yet, a cell line obtained from the natural host of *E. tenella* and from epithelial tissue should be tested alongside mammalian cell lines. As we observed the presence of extracellular merozoites II only when using CLEC-213, we then determined the ability of the parasite to develop further only in this recently characterised cell line [14]. In this avian epithelial cell model, we observed the production of macrogametes and microgametes, using specific markers EtGAM-56 and EtFOA1, respectively [15], thus proving the occurrence of gametogony after infection with the precocious strain and oocyst production after infection with second generation merozoites (merozoites II) of the wild type strain.

## Methods

### Epithelial cell lines

MDBK cells, m-IC_cl2_ cells and CLEC-213 cells were grown as described by Tierney et al. [8], Bens et al. [16] and Esnault et al. [14], respectively. Cells were plated in 24-well plates for the different assays.

### Parasites

Different protocols either in vitro and in vivo were applied to obtain oocysts, sporozoites, merozoites I, merozoites II and gametocytes. To obtain oocysts, 4–6 week-old outbred PA12 White Leghorn chickens were inoculated with 10^4^ sporulated oocysts of *E.tenella* wild type Wisconsin strain (Wis; [17]), wild type recombinant strains expressing the yellow fluorescent protein (Wis-YFP), mCherry strain (INRA-mCherry) or with 10^6^ sporulated oocysts of the precocious *E. tenella* Wis-F-96 strain [6]. Recombinant strains were generated as described by Yan et al. [18]; see below. Chickens were sacrificed after 7 and 5 days post-infection for the wild type strains and the precocious strain, Wis-F-96, respectively. Unsporulated oocysts were collected from the caeca, then sporulated, and sporozoites were purified as described by Shirley [19].

Merozoites I were obtained from supernatants of infected MDBK cells at 72 h p.i. (in vitro) [19]. Merozoites II were obtained from PA12 chickens that had been inoculated orally with 2.5 × 10^5^ sporulated oocysts of *E. tenella* Wis or INRA-m-Cherry 5 days earlier, as described previously [19]. Gametocytes were isolated from caeca of *E. tenella* Wis infected chicken as described previously [19].

### Transgenic *Eimeria tenella*

Recombinant strains were generated using an adaptation of the protocol described by Yan et al. [18]. The Wis strain was transfected with a plasmid expressing the *yfp* gene under the *E. tenella mic1* promoter to allow visualization of parasites in invasion and schizont development assays. The INRA strain PAPt36 [20] was transfected with the *mCherry* gene under the *E. tenella* actin promoter, to facilitate visualization of sexual stages of parasite development in the CLEC-213 cells, where a high background green autofluorescence interferes with detection of YFP signals as parasites developed. Wild type Wis and INRA strains were compared in vivo and did not show, in our experimental conditions with an inoculum of 5000 oocysts, any significant differences in parasite development as shown by oocyst excretion and pathogenicity (lesion scores; data not shown). Similarly, parasites expressing YFP or mCherry protein did not display any differences in parasite development as shown by oocyst excretion (data not shown). Transgenic *E. tenella* oocysts, sporozoites and merozoites II were obtained as described above.

### In vitro parasite development assays

Invasion assays were performed as described previously [21]. Briefly, 2 × 10^5^ epithelial cells per well (MDBK, m-IC_cl2_, CLEC-213) were co-cultured on glass coverslips with 4 × 10^5^ purified sporozoites (Wis-YFP) for 3 h at 41 °C, 5% CO_2_; in preliminary experiments, the infection rate was higher at 41 °C than at 37 °C in all cell lines, confirming the data described by Tierney et al. [8]_._ After washing and fixing the cells with paraformaldehyde (4%; Santa Cruz Biotechnology, Dallas, TX, USA), monolayers were mounted in Vectashield mounting media with DAPI (1.5 μg/ml; Vector Laboratories, Burlingame, CA, USA). For each coverslip, a minimum of three microscope fields were observed (Zeiss Axiovert 200 microscope, Carl Zeiss, Göttingen, Germany). More than 200 cells were counted per condition using ImageJ software. The percentage of infected cells was calculated and values are reported as mean ± SD of at least four independent replicates.

Schizont development was studied by infecting 2 × 10^5^ epithelial cells per well (MDBK, m-IC_cl2_, CLEC-213), cultured overnight on glass coverslips, with 4 × 10^5^ purified sporozoites (Wis-YFP) as described above. After 3 h of co-culture, extracellular sporozoites were removed by washing and cells were kept in culture for an additional 48 h. Cells were washed, fixed and mounted. The percentage of schizonts was calculated relative to the number of infected cells. A minimum of three microscope fields were observed for each coverslip (Zeiss Axiovert 200 microscope, Carl Zeiss); more than 200 cells were counted for each condition. Values are reported as mean ± SD of at least four independent replicates.

The development of sexual stages of *E. tenella* was observed only in CLEC-213 cells (2 × 10^5^ cells per well) infected with either 3 × 10^6^ merozoites II from wild type parasites or 4 × 10^5^ purified sporozoites from the precocious Wis-F-96 in complete cell culture medium for 3 h at 41 °C, 5% CO_2_. After removal of extracellular parasites, cells were cultured for an additional 2 days or 5 days for cells infected with merozoites II or sporozoites, respectively. Then, after washing, cells were fixed with paraformaldehyde (4%; Santa Cruz Biotechnology). The presence of micro and macrogametes was analyzed by specific immunostaining (described below) or by detection of mCherry signal at a wavelength of 620 nm.

### Detection of microgametes and macrogametes in vitro

EtGAM-56 and EtFOA1 antigens, expressed specifically by macrogametes and microgametes, respectively, were detected after staining with specific antibodies (polyclonal rabbit anti-EtGAM-56; 1/100 [15] and monoclonal mouse anti-FOA1 1/100; [15]) and the appropriate secondary antibodies (goat anti-rabbit conjugated with Alexa 594; goat anti-mouse conjugated with Alexa 594; 1/1000; Invitrogen, Eugene, OR, USA). Cell monolayers were mounted as described above and parasite stages were visualised using AxioVision Sofware (Zeiss Axiovert 200 microscope, Carl Zeiss).

In addition to detection using specific antibodies, gene expression of *Etgam56* and *Etfoa1* was studied in different developmental stages of *E. tenella*, after infection of CLEC-213 with merozoites II from wild type strains (Wis) or sporozoites from the precocious strain (Wis-F-96) to confirm the presence of microgametes and macrogametes. Cells or parasites (sporozoites, merozoites I, gametes) were lysed in RA1 lysis buffer (NucleoSpin RNA, Macherey Nagel, Düren, Germany); RNA was extracted as described in the manufacturer’s manual and suspended in nuclease-free water. The Superscript™ II First Strand Synthesis System (Invitrogen), with random hexamer primers and oligo(dT)15 primer (Promega, Madison, WI, USA), was used to synthetize cDNA. For studying the development of gametes, segments of cDNAs were amplified by qPCR (Bio-Rad Chromo4, Bio-Rad, Hercules, CA, USA) using SYBR Green master mix (Bio-Rad), specific primers to the housekeeping gene, *Et18S* (forward primer, 5′-CTG ATG CAT GCA ACG AGT TT-3′ and reverse primer, 5′-GAC CAG CCC CAC AAA GTA AG-3′), the microgamete-specific gene, *Etfoa1* (ETH_00025255 from ToxoDB release 34, www.toxodb.org; forward primer, 5′-TCT CGC ATT CCT CAC AGA TG-3′ and reverse primer, 5′-ATT TCG CCT TGT GGA TGA AC-3′) and the macrogamete-specific gene, *Etgam56* (ETH_00007320 from ToxoDB release 34; forward primer, 5′-AGT GGC TGG AGA ACT TCG TG-3′ and reverse primer, 5′-ATG CGG TTC GTG ATC ATG TC-3′, Eurogentec, Seraing, Belgium). The qPCR was performed using the following protocol: 95 °C for 3 min and 40 cycles at 95 °C for 10 s and 60 °C for 30 s followed by 65 °C for 30 s. The melting curve was generated at 65 °C for 5 s followed by gradual heating (0.5 °C/s) to 95 °C. For each experiment, qPCR were performed in triplicate and expression of *EtGam56* and *Etfoa1* was normalised to Ct values obtained for *E. tenella* small ribosomal subunit *18S* RNA using the formula: 2^-(Ct*gene* – CtE*t18S*)^. Gene expression values are expressed as mean ± SD of at least two independent biological and three technical replicates.

### Statistical analysis

Data were analyzed using ANOVA followed by Dunn’s multiple comparisons test using GraphPad Prism® 6 (GraphPad Software Inc., La Jolla, CA, USA).

## Results

**Fig. 1:**
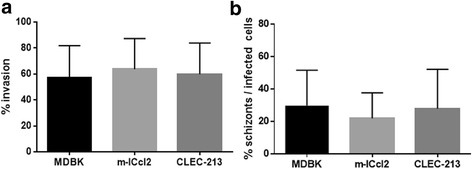
Cell invasion and development to schizonts by *E. tenella* Wis-YFP parasites in MDBK, m-IC_cl2_ and CLEC-213 cell lines. Cells were infected with sporozoites from *E. tenella* Wis-YFP at a multiplicity of infection (MOI) of 2 for 3 h at 41 °C. **a** Cell invasion was calculated as % of cell invasion by sporozoites in different cell lines. **b** Parasite development to the first stage schizont was assessed after 48 h infection and calculated as % of infected cells with schizonts within the total number of infected cells. Data represent the mean ± SD of at least four experiments. No statistically significant difference was found between cell lines (ANOVA)

**Fig. 2:**
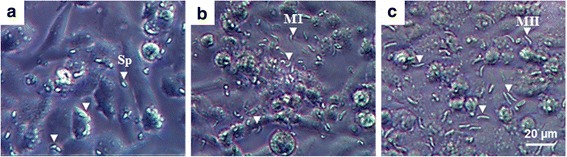
Infection and development of *Eimeria tenella* WIS in CLEC-213. Cells were infected with sporozoites from *E. tenella* Wis at a multiplicity of infection (MOI) of 2 for 3 h at 41 °C. After washing, cell culture was maintained for 1 day (**a**), 3 days (**b**) or 4 days (**c**) p.i. and observed using bright-field microscopy. White arrowheads show (**a**) intracellular sporozoites (trophozoites), (**b**) first generation merozoites and (**c**) second generation merozoites released into the medium. *Abbreviations*: Sp, Sporozoites; MI, first generation merozoites; MII, second generation merozoites. *Scale-bar*: 20 μm

We compared parasite invasion and development to schizonts in different cell lines (MDBK, m-IC_cl2_ and CLEC-213). For all cell lines, we observed similar percentages (~60%) of parasite invasion (Fig. 1a). We assessed the ability of the parasite to develop further by counting the number of schizonts I after 48 h of infection; we found that 20–30% of infected cells in all cell lines harboured schizonts (Fig. 1b). For all cell lines, merozoites I were found in the cell culture medium at 72 h p.i.; there was no merogony II when using MDBK or m-IC_cl2._ However, extracellular merozoites II were observed in CLEC-213 cells (Fig. 2c) suggesting the possibility for further parasite development with this specific cell line.

**Fig. 3:**
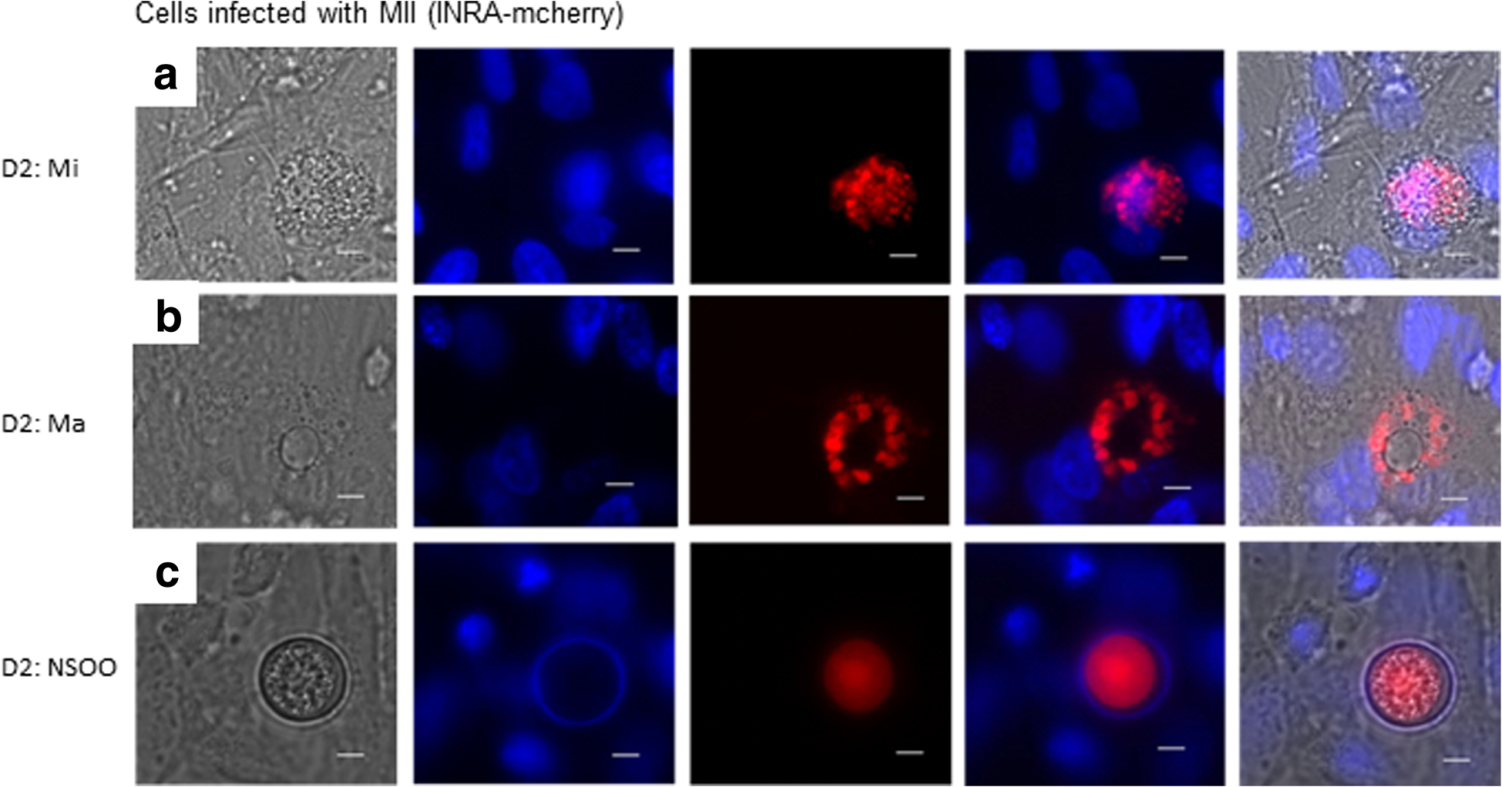
Sexual development of *Eimeria tenella* INRA-mCherry parasites in the chicken epithelial cell line, CLEC-213. Cells were infected with merozoites II from *E. tenella* INRA-mCherry. Two days p.i., parasites were visualised by m-Cherry protein expression and cell nuclei were visualized by DAPI counterstaining using fluorescent microscopy. Pictures are representative of at least two experiments. *Abbreviations*: Mi, microgametes (**a**); Ma, macrogametes (**b**) NSOO, non-sporulated oocysts (**c**). *Scale-bars*: 5 μm

**Fig. 4:**
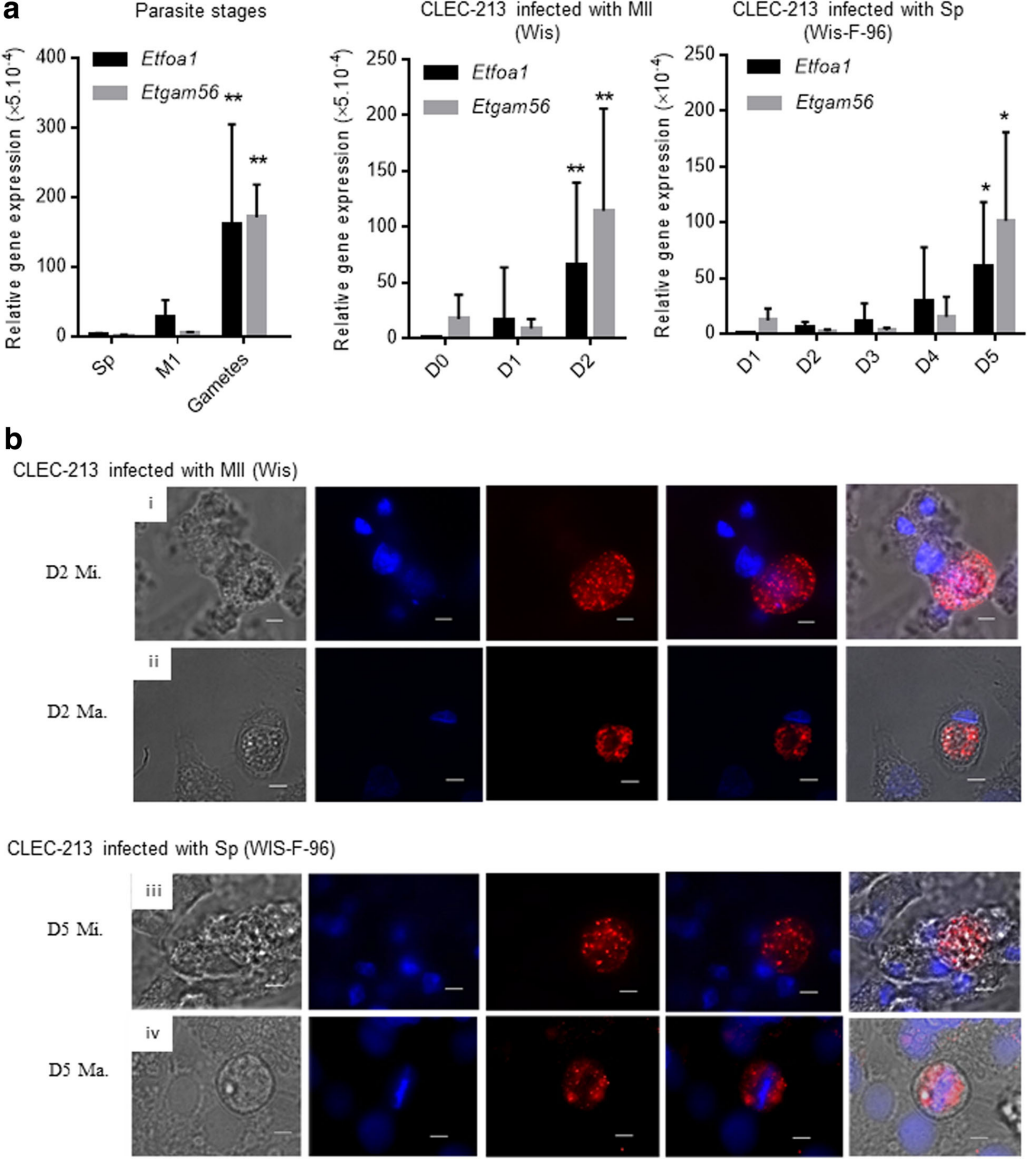
Relative gene expression and localization of the *Eimeria tenella* FOA1 and GAM-56 proteins in parasites grown in the chicken epithelial cell line, CLEC-213. **a** Relative gene expression of *Etfoa1* and *Etgam56* during parasite development stages. *Left panel*: RT-qPCR was performed on different enriched parasite stages of *E. tenella*: sporozoites (Sp) obtained by excystation, merozoites (M1) obtained from in vitro culture and gametes purified from caeca from infected chicken. *Middle panel*: RT-qPCR was performed on CLEC-213 infected with merozoites II from *E. tenella* wild type Wis strain (day 0 to day 2 p.i.). *Right panel*: RT-qPCR was carried out on CLEC-213 infected with sporozoites from the precocious strain of *E. tenella,* Wis-F-96 (day 1 to day 5 p.i.). The relative transcript expression of *Etgam56* and *Etfoa1* was defined relative to the *E. tenella* housekeeping gene, *Et18S.* Statistical analysis was performed using ANOVA followed by Dunn’s test. *P < 0.05 and **P < 0.01 indicate a statistically significant difference compared to previous free parasite stages (Sp and M1) or previous days of infection. Data are the mean ± SD of at least two biological replicates and three technical replicates. **b** Immunofluorescence analysis of microgametocytes and macrogametocytes production in CLEC-213 infected with merozoites II from *E. tenella* Wis strain (i, ii) or sporozoites from the precocious Wis-F-96 strain (iii, iv) was performed using anti-FOA1 mouse sera (i, iii) and anti-GAM56 rabbit sera (ii, iv) and the appropriate secondary antibodies (Alexa 594). Monolayers were mounted with DAPI counterstaining and parasites were visualised by fluorescent microscopy. *Abbreviations*: Sp, Sporozoites; MI, first generation merozoites; MII, second generation merozoites, Mi, microgametes (i, iii); Ma, macrogametes (ii, iv). *Scale-bars*: 5 μm

In order to increase the probability of gamete formation, CLEC-213 were infected with merozoites II isolated from the caeca of infected chickens. Merozoites II (INRA-mCherry) were able to invade the cells. After 2 days of infection, the presence of forms suggesting the presence of microgametes, macrogametes and unsporulated oocysts was observed by microscopy (Fig. 3). To confirm this observation, we used specific markers described by Walker et al. [15]: EtGAM-56, a macrogamete specific protein incorporated into the oocyst wall and EtFOA1, a flagellar outer arm protein (also known as a dynein intermediate chain protein), which is specific to microgametes. First, we confirmed the increase of *Etfoa1* and *Etgam56* gene expression in purified gametes compared to sporozoites or merozoites I obtained in vivo as described by Walker et al. [15]. Then, CLEC-213 were infected with merozoites II from the wild-type Wis strain of *E. tenella,* and *Etfoa1* and *Etgam56* gene expression levels were measured by RT-qPCR. Additionally, EtFOA1 and EtGAM56 protein expression was detected by immunofluorescence. In these conditions, an increase in *Etfoa1* and *Etgam56* gene expression was observed compared to cells at previous time points (Fig. 4a); this observation was confirmed by the detection of forms that were revealed by using specific antibodies for EtFOA1 and EtGAM56 suggesting the presence of microgametes and macrogametes, respectively (Fig. 4b (i, ii)).

Maintaining CLEC-213 in culture for 7 days can be challenging. We therefore attempted to replicate our results using sporozoites from the precocious strain, Wis-F-96, which displays a shorter parasite cycle than the wild type strain (7 days versus 5 days in vivo [6]). In these experimental conditions, we were again able to demonstrate an increase in *Etfoa1* and *Etgam56* gene expression, this time at day 5 post-infection (Fig. 4a), and were able to confirm the presence of microgametes and macrogametes by immunofluorescence (Fig. 4b (iii, iv)). However, we were unable to observe any oocysts after infection of CLEC-213 cells with sporozoites of WIS-F-96, though we cannot rule out that they were present at levels below our ability to detect at this earlier time-point.

## Discussion

*Eimeria tenella* is able to invade many different cell lines in vitro, as shown by Tierney et al. [8]. However, the parasite’s life-cycle has been observed to proceed to completion, in vitro only in chick embryonic kidney cells or primary chicken kidney cells (PCKC), where sexual stages (i.e. macrogametes and microgametes) as well as oocysts have been observed after infection with sporozoites [7, 12, 22–25]. The complete life-cycle has also been achieved by *in ovo* infection of chorioallantoic membranes of chick embryos [22]. In most currently tested cell lines, *E. tenella* develops only asexually, halting at the first generation of schizont in cultures of mammalian fibroblasts, mammalian epithelial cells, and avian fibroblasts [8, 26]. In vitro invasion of chick embryo kidney, chick embryo fibroblast, mouse fibroblast, human amnion, and HeLa cell cultures by *E. acervulina* sporozoites do not lead to further parasite development [27]. However, studies using, non-poultry species of *Eimeria* (e.g. *E. bovis*, *E. ovinoidalis*, *E. ninakohlyakimovae*) have shown that the parasite can develop to various stages (merozoites I and a few gametocyte-like stages) in several cell types, including bovine, human and porcine endothelial cell lines, bovine foetal gastrointestinal cells, bovine kidney epithelial cells and African green monkey kidney epithelial cells [28–32]. More particularly, the development of *E. nieschulzi* macrogamonts and oocysts were observed when cultured with mixed cells derived from inner fetal organs [32]. Here, we show similar levels of parasite invasion and development to the first generation of schizont in bovine epithelial-like kidney (MDBK), murine intestinal epithelial (m-IC_cl2_) and avian epithelial (CLEC-213) cells. For all cell lines, merozoites I were observed in the cell culture medium at 72 h p.i. but no extracellular merozoites II were observed when using MDBK or m-IC_cl2_ cells. These observations confirmed previous data using MDBK cells [8–11, 26] and, furthermore, demonstrated that a mammalian intestinal epithelial cell line (m-IC_cl2_) and an avian, non-intestinal epithelial cell line (CLEC-213) were at least equally permissive to invasion and initial asexual reproduction by *E. tenella*. However, in our experimental conditions, extracellular merozoites II were observed only in CLEC-213 cells, suggesting the possibility for further parasite development in this specific chicken epithelial cell line.

In order to maximize the chance of achieving development of sexual stages of *E. tenella* in vitro and minimize the time taken to reach these stages of development, we implemented two strategies: first, we infected CLEC-13 cells with purified merozoites II harvested from infected caeca and secondly, we infected CLEC-213 cells with sporozoites of a strain of *E. tenella* (Wis-F-96) that enters gametogony after only a single asexual cycle. In both cases, we observed forms consistent with microgametes and macrogametes, within two days of infection of cells with wild type merozoites II and within five days of infection with the precocious-strain sporozoites. To confirm the characterization of the putative gametes, we used specific markers described by Walker et al. [15]. Briefly, EtGAM-56 is a macrogamete specific protein that is destined for incorporation into the oocyst wall. EtGAM-56 co-localises to wall forming bodies that are characterised by their doughnut-like appearance. EtFOA1 is a flagellar outer arm protein 1, also known as a dynein intermediate chain protein that is specific to microgametes [15]. We studied the development of the parasite by measuring gene expression of *Etfoa1* and *Etgam56* and we confirmed that these genes are mainly expressed in gametes as shown in enriched gamete fractions compared to the free stages of the parasite (sporozoite, Sp, and merozoites I, M1). We then demonstrated the presence of gametes when cells were infected with merozoites II, 2 days p.i. as shown both by an increase in *Etfoa1* and *Etgam56* gene expression and by protein detection using specific antibodies. When cells were infected with sporozoites from the precocious strain for five days, we also observed an increase in gamete gene expression and we again detected the presence of gametes with specific antibodies. Additionally, we were able to observe fully-formed, unsporulated oocysts in the CLEC-213 cell cultures originally infected with the wild type merozoites II, demonstrating that completion of the final steps of the parasite cycle is possible in CLEC-213 cells. We also infected CLEC-213 cells with *E. tenella* Wis wild type strain; however, because the reproductive cycle of this parasite strain is longer than that for the precocious strain [6], we did not consistently observe sexual stages. It cannot be ruled out that optimisation of culture conditions for these cells may allow sexual development of the wild type parasite.

## Conclusions

Thus overall, we have demonstrated for the first time, when using sporozoites from a precocious strain or merozoites II from a wild type strain, that in CLEC-213, a chicken epithelial cell line permits the development of sexual stages - both microgametes and macrogametes - of the parasite *E. tenella* in vitro, resulting in production of oocysts. Although, the infected culture leads to only relatively low yields of gametes, it is important to note that they can be detected and relative burden assessed easily by qPCR. Thus, this new avian epithelial cell model of infection holds great promise and constitutes a first step towards the development of a new tool for drug screening, and for assessment of the effect of immune factors on the *Eimeria* life-cycle, as it is now possible to carry out investigations on both asexual and sexual stages in vitro.

## Abbreviations

CLEC-213: Chicken lung epithelial cells
FOA1: Flagellar outer arm protein 1
GAM-56: Macrogamete specific protein
M1: First generation merozoites
Ma: Macrogametes
MDBK: Madin-Darby bovine kidney epithelial cells
Mi: Microgametes
m-IC_cl2_: Transimmortalized mouse intestinal cells
MII: Second generation merozoites
NSOO: Non-sporulated oocysts
PCKC: Primary chicken kidney cells
Sp: Sporozoites
Wis: Wisconsin
YFP: Yellow fluorescent protein
